# Regulatory polymorphisms in extracellular matrix protease genes and susceptibility to rheumatoid arthritis: a case-control study

**DOI:** 10.1186/ar1849

**Published:** 2005-11-01

**Authors:** Julio Rodriguez-Lopez, Eva Perez-Pampin, Juan J Gomez-Reino, Antonio Gonzalez

**Affiliations:** 1 Research Laboratory 2 and Rheumatology Unit, Hospital Clinico Universitario de Santiago, Santiago de Compostela, Spain; 2 Department of Medicine, University of Santiago de Compostela, Santiago de Compostela, Spain

## Abstract

Many extracellular matrix (ECM) proteases seem to be important in rheumatoid arthritis (RA) and regulation of their transcription levels is a critical mechanism for controlling their activity. We have investigated, therefore, whether the best-characterized single nucleotide polymorphisms (SNPs) affecting transcription of the ECM proteases that have been related with joint pathology are associated with RA susceptibility. Nine SNPs in eight genes were selected by bibliographic search, including SNPs in the genes encoding matrix metalloproteinase (MMP)1, MMP2, MMP3, MMP7, MMP9, MMP13, plasminogen activator, tissue type (PLAT) and PAI-1. They were studied in a case-control setting that included 550 RA patients and 652 controls of Spanish ancestry from a single center. Genotyping was performed by single-base extension. Only two of the nine SNPs showed significant association with RA susceptibility. RA patients showed increased frequencies of the -7351 T allele of the gene encoding PLAT (36.4% versus 32.1% in controls, *p* = 0.026) and the -1306 T allele of the gene encoding MMP2 (24.5% versus 20.3% in controls, *p* = 0.013). These two alleles seemed to cooperate according to an additive model with respect to increased RA susceptibility (*p* = 0.004), and they were the low-expression alleles of the respective SNPs in a *PLAT *enhancer and the *MMP2 *promoter. These findings are in agreement with previous data suggesting that these two ECM proteases have a protective role in RA pathology. Confirmation of these associations will be needed to support these hypotheses. The remaining SNPs did not show association, either individually or collectively. Therefore, although regulatory SNPs in ECM proteases did not show any major effect on RA susceptibility, it was possible to find modest associations that, if replicated, will have interesting implications in the understanding of RA pathology.

## Introduction

Many studies support an important role for genetic factors in rheumatoid arthritis (RA) susceptibility and progression [1]. Overall, the genetic component has been estimated to account for about 50% of the variance in disease susceptibility, the remainder being environmental and stochastic components. The best known RA genetic factor is the human leukocyte antigen (*HLA*) gene, where multiple alleles of the DRbeta1 chain that share a common epitope in the third hypervariable region determine disease susceptibility and severity. Other HLA molecules and several non-*HLA *genes have also been related with RA susceptibility. Among the many genes that have been studied, only two that encode extracellular matrix (ECM) proteases have been explored [2-6], despite the unequivocal involvement of this family of proteins in RA.

The ECM proteases comprise a large family of proteins grouped in several subfamilies, including the matrix metalloproteinases (MMPs), the most extensively studied in RA [7-9]. Many MMPs are expressed at increased levels in RA tissues and in synoviocyte cultures in response to inflammatory cytokines, show specificity for joint tissue components and affect the evolution of experimental models of arthritis. Drugs able to inhibit a wide array of MMPs have been tried for the treatment of RA and, although effective in experimental models, human clinical trials had to be discontinued due to intolerable side effects. It is expected that more specific protease inhibitors will retain therapeutic potential without the associated side effects. It is unclear what ECM proteases to target with these drugs, however, because it has been difficult to ascertain the specific participation of each of them in RA. As a group, they are the major actors in the degradation of ECM in RA cartilage and bone. In addition, they increase and perpetuate joint inflammation through the activation of cytokines, chemokines and other proteases by cleavage of their precursors at specific sites [7-10]. They can also contribute to inflammation by exposing cryptic epitopes in ECM components that have biological actions in angiogenesis, cell migration and proliferation [10]. The difficulty in discerning the specific role of each of the ECM proteases found in the joints stems from the apparent redundancy of their effects and the wide variety of targets that each could degrade. We expect that genetic studies will provide clues to the identities of proteases that are critical for the RA process.

We have searched bibliographic databases for ECM proteases and their specific inhibitor proteins that have been described as involved in cartilage homeostasis and joint pathology. About 35 were identified with very varied supporting evidence, including some with a putative protective effect. We looked for evidence of single nucleotide polymorphisms (SNPs) in the genes encoding these ECM proteases that have shown a regulatory effect on their transcription level, most often from reporter gene assays but also from electrophoretic mobility-shift assays and in some cases from *ex vivo *studies. Nine SNPs in eight genes that fulfilled these criteria were found. In addition, each of these SNPs has been associated with susceptibility to at least one from a wide list of diseases, including cardiovascular diseases, aneurysms, preterm rupture of amniotic membranes, and tumor metastasis, indicating that their effects in gene transcription have a significant *in vivo *repercussion. The nine SNPs were found in the promoters or enhancers of the genes encoding MMP1 [11], MMP2 [12], MMP3 [13], MMP7 (with two SNPs) [14], MMP9 [15], MMP13 [16], plasminogen activator, tissue type (PLAT) [17] and plasminogen activator inhibitor-1 (PAI-1) [18]. They were selected as appropriate candidates to participate in RA susceptibility and studied in a large case-control setting. Two of the SNPs, those in *PLAT *and *MMP2*, showed moderate association with RA. The alleles of these two SNPs found with increased frequency in RA patients are low-expression alleles, which is in agreement with previous data suggesting that these ECM proteases have a protective effect in RA.

## Materials and methods

### Patients and controls

We sought to include all the 980 RA patients followed in the Rheumatology Unit of the University Clinical Hospital of Santiago de Compostela. Of these, 91 patients were not retrievable, 98 had died or were too sick to participate and 85 were unwilling to collaborate. Of the remaining 706 patients, 156 were excluded because they had non-Spanish ancestry or because of discrepancies with the American College of Rheumatology revised classification criteria for RA; 550 RA patients were available for the study. The control samples were from 652 subjects older than 55 years of age undergoing preoperative work-up for elective surgery excluding orthopedics. All were of Spanish ancestry and resided in the reference area of the Hospital. The Ethical Committee for Clinical Research of Galicia approved this study and all participants gave their written informed consent.

### Genotyping

Peripheral blood DNA was used to genotype the following nine SNPs: MMP1 -1607 1G/2G (rs1799750); MMP2 -1306 C/T (rs243865); MMP3 -1171 6A/5A (rs3025058); MMP7 -181 A/G and -153 C/T; MMP9 -1562 C/T (rs3918242); MMP13 -77 A/G (rs2252070); PLAT -7351 C/T (rs2020918); and PAI-1 -675 5G/4G (rs1799768).

PCR was performed in two multiplex reactions with the QIAGEN Multiplex PCR Kit (QIAGEN, Valencia, CA, USA), each containing 30 ng of genomic DNA. One multiplex reaction was carried out for PLAT, MMP13, PAI, MMP9 and MMP2, and the other included MMP3, MMP7 and MMP1. PCR conditions were: initial denaturation at 95°C for 15 minutes, followed by 35 cycles of denaturation at 94°C for 30 s, annealing at 60°C for 90 s, and extension at 72°C for 90 s. Final extension was performed for 10 minutes at 72°C. Primers were designed with the FastPCR software (obtained from Dr Ruslan Kalendar, University of Helsinki). PCR products were purified by Exo-SAP digestion with Exonuclease I (Epicentre, Madison, WI, USA) and shrimp alkaline phosphatase (SAP; Amersham Biosciences, Barcelona, Spain) for 1 h at 37°C, and 15 minutes at 75°C to inactivate the enzymes. Single-base extension reactions with the SNaPshot Multiplex Kit (Applied Biosystems, Foster City, CA, USA) were done. Reaction conditions were: 25 cycles of denaturation at 96°C for 10 s, annealing at 50°C for 5 s and single-base extension at 60°C for 30 s. Post-extension treatment with SAP was done for 1 h at 37°C. Samples were analyzed in the ABI prism 3100 Avant Genetic Analyzer (Applied Biosystems). Sequences of the PCR primers and of the single base extension oligonucleotides are available from the authors upon request.

### Sequencing

Several samples with each of the observed genotypes were sequenced to test the accuracy of genotyping. The system used for sequencing was the Big Dye Ready Reaction Kit (Applied Biosystems) on an ABI prism 3100 Avant Genetic Analyzer (Applied Biosystems). Cycling conditions were: initial denaturation at 96°C for 4 minutes, followed by 30 cycles of denaturation at 96°C for 15 s, annealing at 50°C for 10 s, and extension at 60°C for 3 minutes. Final elongation was done at 60°C for 10 minutes.

### Statistical and genetic analysis

Statistical analysis was done with the Statistica software (Statsoft, Tulsa, OK, USA). Allele frequencies, their interquartile ranges (IQR), odds ratios (ORs) and their 95% confidence intervals (95% CI) were calculated. Comparison of allele frequencies was done using a two by two contingency table with a chi-square test. Evidence of a gene dose effect was evaluated with univariate logistic regression applying an additive genetic model (codes were: 0 for AA, 1 for Aa and 2 for aa genotypes). Multivariate comparison of the coordinate effect of the protease genotypes, as well as analysis of the effect of clinical features as covariants, was done with stepwise backward logistic regression analysis. Analysis of the gene-gene interaction between the *PLAT *and *MMP2 *SNPs was done with the LRASSOC software [19], which implements well-defined genetic models for gene interactions. Model selection was based in the lowest Akaike's information criterion. *Post hoc *power of the study was estimated for alfa = 0.05 with the Gpower software [20]. Relationships between clinical features and genotypes were analyzed with Student's *t* test for quantitative variables and chi-squared test for the contingency tables of qualitative features.

Linkage disequilibrium (LD) between the SNPs in chromosome 11 was analyzed with the *ldmax *software [21]. Haplotype frequencies were estimated with the PL-EM software [22], which uses an implementation of the expectation-maximization algorithm. Comparison of haplotype frequencies was done with a nonparametric homogeneity test and with a permutation test performed with the Clump software [23].

## Results

### Study characteristics

The characteristics of the RA patients are shown in Table 1. Women were more abundant in the RA group (421 of 550, 76.5%, 95% CI = 73–80) than in the control group (344 of the 642, 52.8%, 95% CI = 49–56). This difference did not affect the results, however, as associations were independent of sex as shown below. The median age at disease onset was 49 years (IQR 37–57) and the median follow-up was 13 years (IQR 7–21 years). Controls (median age = 69 years, IQR 62–76 years) were selected over 55 years of age that corresponded to percentile 70 of the age at disease onset in our series of RA patients. Genotypes for the nine SNPs analyzed were determined unambiguously in 99.7% of the samples, and confirmed by sequencing a fraction of them. The genotype distributions of all SNPs were in concordance with the Hardy-Weinberg equilibrium.

### Genetic susceptibility to RA

**Figure 1:**
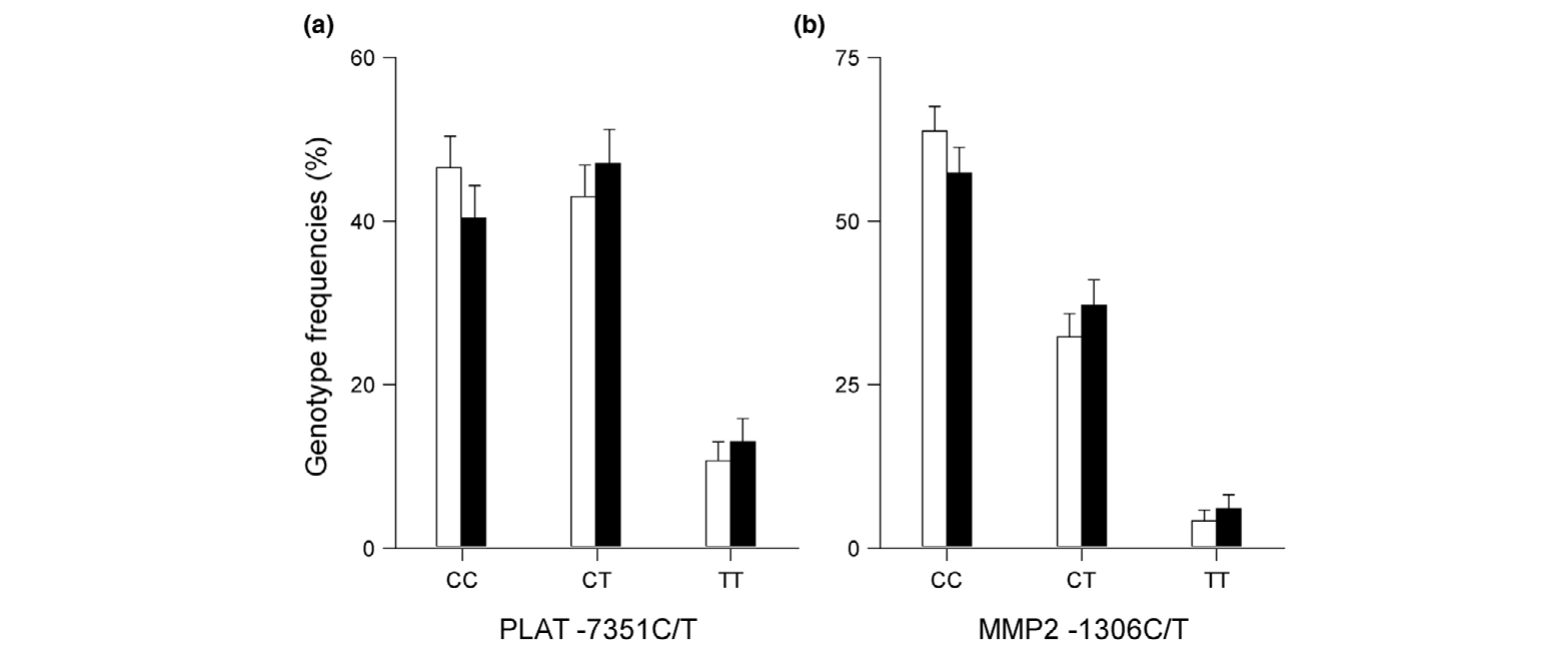
Genotype frequencies (%) of single nucleotide polymorphisms (SNPs) associated with rheumatoid arthritis (RA). **(a) **The *PLAT *-7351 C/T and **(b) **the *MMP2 *-1306 C/T SNP in controls (blank columns) and RA patients (filled columns). Error bars represent the 95% confidence interval.

Allelic frequencies of seven of the nine SNPs were similar in RA patients and controls (Table 2). Only the *PLAT *-7351 C/T SNP and the *MMP2 *-1306 C/T SNP were significantly different. In the case of *PLAT*, the T allele was significantly (*p* = 0.026) more frequent in the RA patients (36.4%, 95% CI = 33–39) than in controls (32.1%, 95% CI = 29–34). In the case of *MMP2*, the T allele was significantly (*p* = 0.013) more frequent in RA patients (24.5%, 95% CI = 22–27) than in controls (20.3%, 95% CI = 18–22). Analysis of genotype frequencies by univariate logistic regression produced similar results: the effect of the T allele of *PLAT *-7351 C/T was dose-dependent according to an additive genetic model (*p* = 0.026; OR = 1.21, 95% CI 1.02–1.43) as shown in Figure 1a; similarly, the effect of the T allele of *MMP2 *-1306 C/T was in agreement with an additive genetic model (*p* = 0.013; OR = 1.27, 95% CI 1.05–1.55) as shown in Figure 1b. These results were not significantly modified by the inclusion of sex as a covariant (Table 2): the OR for *PLAT *genotypes was 1.20 after adjusting for sex and the OR for *MMP2 *was unchanged after the inclusion of this covariant (OR = 1.27). Similarly, the sex-adjusted ORs of the other ECM protease SNPs were not significantly different from the unadjusted ORs (Table 2).

Gene-gene interactions that could involve any of the nine ECM protease SNPs were ascertained with multivariate logistic regression analysis. Only an additive genetic model was tested. The inclusion of the different SNPs was based on a backwards stepwise approach. The best model included only the two previously mentioned SNPs (*PLAT *-7351 C/T and *MMP2 *-1306 C/T) and it showed a slightly better fit to data than any of the two SNPs separately (*p* for the model with the two SNPs = 0.004). There was no evidence of significant gene-gene interactions with any of the other seven SNPs, those without an effect in the individual analyses. We also explored whether there were any specific relationships between the clinical features of the RA patients (Table 1) and the nine SNPs, but none was found.

The statistical characteristics of the interaction between the *PLAT *and *MMP2 *SNPs were analyzed with the LRASSOC software. This software checks the relative fitting to data of a series of genetic models that include parameters for additive, dominance and epistatic interactions between two genes. The model that best accounted for the data was the model assuming additive effects of both SNPs, without dominance or interactive components.

Five of the studied ECM protease genes (*MMP7*, *MMP1*, *MMP3 *and *MMP13*) are in a MMP cluster that covers 500 kb in chromosome 11q and includes at least another five MMP-encoding genes. Therefore, we checked if they were in LD and if the haplotypes defined by them were associated with RA as a way to explore possible effects in the region not accounted for by the studied SNPs. There was significant LD between the two SNPs in *MMP7 *(-181 A/G and -153 C/T), between the *MMP1 *-1607 1G/2G and *MMP3 *-1171 6A/5A SNPs, and between *MMP13 *-77 A/G and two other SNPs (*MMP3 *-1171 6A/5A and *MMP7 *-153 C/T). Comparison of the frequencies of the haplotypes defined by the pairs of SNPs in LD did not disclose significant differences between RA patients and controls (not shown).

## Discussion

The lack of association with RA of seven of the regulatory SNPs in ECM proteases was somehow unexpected because they affect proteases with a recognized role in RA [7-9] and because the size of the study allowed for detection of modest effects (for example, the *post hoc *power to detect an excess of the T allele of the *MMP9 *SNP was 87% for a risk ratio of 1.2). In fact, there is much more published evidence supporting the involvement of some of the ECM proteases that did not show association in our study than the two that were associated with RA. This is especially clear for the metalloproteinases MMP13, MMP1, MMP3 and MMP9. We also analyzed the possibility of cooperation or of cumulative effects between these SNPs with regard to their association with RA. It would be a mistake, however, to interpret too strongly these results as questioning the importance of these six ECM proteases in RA. At least two factors moderate a conclusion of this type. The multiplicity of control mechanisms of ECM protease activity, of which transcription regulation is only one of importance, compartmentalization by pericellular accumulation, activation by cleavage of latent pro-enzymes and inhibition by specific proteins being the others [7,8]. Also, variation in these proteases could impinge on other aspects of disease progression different from disease susceptibility, although we did not find significant association with any of the clinical features available for study. They did not include disease activity indexes or quantitative assessment of bone erosions, however, which could be more informative of the possible involvement of these SNPs. This seems the case for the SNP in *MMP3 *found in previous studies to be associated with quantitatively evaluated RA erosions [3,5], but not to RA susceptibility [3,6]. It is also possible that the SNP in *MMP1 *predisposes to some RA features because there are reports of association with RA inflammatory activity [4] or with RA erosions [6] although these associations were not found in other studies [2].

The two regulatory SNPs that showed moderate association with RA susceptibility cause lower transcription of their respective genes, *PLAT *and *MMP2*. Both of them seem genuine associations because they have been found in a hypothesis driven case-control study and have shown modest effects, in the range that is expected in complex diseases and specifically in RA [1]. The size of the study, 550 cases and 642 controls, effectively prevents interference from random variation of allelic frequencies, which is the major cause of false positive results in genetic association studies [24]. In this regard, it is reassuring that about 90% of the associations shown in studies involving more than 150 cases and controls have subsequently been replicated, as pointed out in a recent meta-analysis [24]. Finally, population stratification, another widely claimed cause of spurious association results, is not a significant concern in this study as all cases and controls comprised a very homogenous population. They resided in a largely rural area where immigration has been very restricted. Specifically, 71.8% of the RA patients and 74.0% of the controls had all known ancestors from the same province (Corunna), and 95.4% of the RA patients and 95.3% of the controls were from the same historic region (Galicia, composed of four of the 52 Spanish provinces). In addition, analysis of data restricted to study participants with all known ancestors from Galicia or from Corunna gave similar results to those from the whole study. Nevertheless, circumspection should be exercised in interpreting these associations until the results can be replicated.

PLAT participation in RA has been related to fibrin accumulation in RA synovial cavities [25-27] and the association found here could be involved in this process. A contributing factor in the increase of fibrin could be the lower or unchanged expression of PLAT in RA synovium compared to healthy synovium [28,29], which would cause the availability of plasmin, the major fibrinolytic enzyme, to be restricted. A putative protective role for PLAT in arthritis has also been shown in experimental models of RA [30,31]. As the T allele of *PLAT *-7351 disrupts a GC box in the *PLAT *enhancer [17,32] it could contribute to the insufficient fibrinolysis and, thus, to RA. Other processes in which hypofibrinolysis is a contributing disease mechanism, such as myocardial infarction [33] and lacunar stroke [34], have also been associated with the T allele of the *PLAT *-7351 C/T SNP and these two diseases are observed at increased rate in RA.

Very few studies have addressed the role of MMP2 in RA. It has been assumed that MMP2 promotes RA by participating in cartilage degradation and by activating pro-inflammatory mediators based on its *in vitro *reactivity [8,9]. However, MMP2 levels and activity seem to be unaltered in human RA [35-37] and in experimental models of RA [38]. In addition, MMP2 plays a suppressive role in the pathogenesis of antibody-induced arthritis, as shown by an exacerbated disease in MMP2 deficient mice [39]. A likely mechanism for MMP2-mediated protection against RA involves the inactivation of chemokines (CCL7 and SDF1), thereby limiting inflammatory infiltration [40,41]. Consistent with a protective role for MMP2 in RA, the allele associated with increased RA susceptibility in our study is the low-expression allele [42,43]. The *in vivo *relevance of this change has been demonstrated by the association of the *MMP2 *-1306 C/T SNP with several types of cancer [43-48], although a recent report showed no association with chronic periodontitis [49], a disease with many similarities to RA with respect to inflammation and ECM proteases [50].

Our study indicates that both SNPs act independently in their contribution to RA liability. This conclusion is consistent with the independent roles proposed for the two proteases for their putative protective effect in RA. Interaction with other gene polymorphisms should be explored as it is likely that variants of cytokine genes that are important in RA and that trigger ECM protease gene expression, especially tumor necrosis factor and interleukin-1 but also interleukin-6, epidermal growth factor, platelet-derived growth factor, basic fibroblast growth factor and transforming growth factor-beta, potentiate the effect of the SNPs studied here. In the same way, it is possible that variants of the genes encoding chemokines that are cleaved by MMP2 could interact with the -1306 C/T SNP in determining increased RA susceptibility.

## Conclusion

It seems that genetic variants affecting transcription of ECM proteases are not major contributors to RA susceptibility. It is possible, however, that some play a minor role as shown here for the *PLAT *and *MMP2 *SNPs. These associations need to be confirmed, although it is already possible to see that the likely effects of these SNPs are consistent with previous evidence supporting a protective effect of the two proteases in arthritis. Confirmation of these associations will lend support to this hypothesis and will show how important it is to define the participation of each ECM protease in joint pathology before trying to manipulate them therapeutically.

## Abbreviations

CI = confidence interval; ECM = extracellular matrix; IQR = interquartile range; LD = linkage disequilibrium; MMP = matrix metalloproteinase; OR = odds ratio; PAI = plasminogen activator inhibitor; PCR = polymerase chain reaction; PLAT = plasminogen activator, PLAU: plasminogen activator, urokinase tissue type; RA = rheumatoid arthritis; SAP = shrimp alkaline phosphatase; SNP = single nucleotide polymorphism.

## Competing interests

The authors declare that they have no competing interests.

## Authors' contributions

All authors contributed to the final manuscript. In addition, JR-L did the genotyping and participated in the design of the study and the analysis of results, EP-P reviewed all the clinical data from the patients and selected them according to the ACR classification criteria, JG-R participated in the selection of patients, and AG coordinated the study and participated in its design, experiments, analysis and in the writing of this manuscript.

## Figures and Tables

**Table 1:** Clinical characteristics of the rheumatoid arthritis patients included in the study

Characteristic	Value
Females (%)	421 (76.5)
Age of disease onset (median, IQR)	49 (37–57)
Follow-up (median, IQR)	13 (7–21)
Rheumatoid factor (%)^a^	280 (62.5)
Bone erosions (%)^a^	284 (64.8)
Extra-articular manifestations (%)^a^	48 (11.2)
DMARDS use (%)^a^	465 (94.9)
Tobacco use (%)^a^	84 (18.4)

^a^This information was not available for a variable number of patients (from 10.9 for disease-modifying anti-rheumatic drugs (DMARDS) use to 21.8% for extra-articular manifestations). IQR, interquartile range.

**Table 2:** Allele frequencies of the nine regulatory single nucleotide polymorphisms in extracellular matrix proteases

Gene	SNP	Allele	RA patients	Controls	OR	*p*	Sex-adjusted OR	Change in transcription^a^
*MMP1*	-1607 1G/2G	2G	47.8	48.2	0.98	0.8	0.96	Up
*MMP2*	-1306 C/T	T	24.5	20.3	1.27	0.01	1.27	Down
*MMP3*	-1171 6A/5A	5A	50.5	51.7	0.95	0.5	0.96	Up
*MMP7*	-181 A/G	G	43.8	45.3	0.94	0.4	0.95	Up
	-153 C/T	T	5.0	5.1	0.99	0.9	0.98	Up
*MMP9*	-1562 C/T	T	14.3	13.6	1.05	0.6	1.02	Up
*MMP13*	-77 A/G	A	68.0	65.6	1.13	0.2	1.09	Up
*PLAT*	-7351 C/T	T	36.4	32.1	1.21	0.03	1.20	Down
*PAI-1*	-675 5G/4G	4G	47.6	46.8	1.03	0.7	1.05	Up

Frequency values for rheumatoid arthritis (RA) and controls are percentages.

^a^Reported up- or downregulation of gene expression determined by the mentioned allele. MMP, matrix metalloproteinase; OR, odds ratio; PAI, plasminogen activator inhibitor; PLAT, plasminogen activator, tissue type; SNP, single nucleotide polymorphism.

## References

[B1] Harney S, Wordsworth BP (2002). Genetic epidemiology of rheumatoid arthritis. Tissue Antigens.

[B2] Constantin A, Lauwers-Cances V, Navaux F, Abbal M, van Meerwijk J, Mazieres B, Cambon-Thomsen A, Cantagrel A (2002). Collagenase-1 (MMP-1) and HLA-DRB1 gene polymorphisms in rheumatoid arthritis: a prospective longitudinal study. J Rheumatol.

[B3] Constantin A, Lauwers-Cances V, Navaux F, Abbal M, van Meerwijk J, Mazieres B, Cambon-Thomsen A, Cantagrel A (2002). Stromelysin 1 (matrix metalloproteinase 3) and HLA-DRB1 gene polymorphisms: Association with severity and progression of rheumatoid arthritis in a prospective study. Arthritis Rheum.

[B4] Lee YH, Kim HJ, Rho YH, Choi SJ, Ji JD, Song GG (2003). Functional polymorphisms in matrix metalloproteinase-1 and monocyte chemoattractant protein-1 and rheumatoid arthritis. Scand J Rheumatol.

[B5] Mattey DL, Nixon NB, Dawes PT, Ollier WE, Hajeer AH (2004). Association of matrix metalloproteinase 3 promoter genotype with disease outcome in rheumatoid arthritis. Genes Immun.

[B6] Dorr S, Lechtenbohmer N, Rau R, Herborn G, Wagner U, Muller-Myhsok B, Hansmann I, Keyszer G (2004). Association of a specific haplotype across the genes MMP1 and MMP3 with radiographic joint destruction in rheumatoid arthritis. Arthritis Res Ther.

[B7] Martel-Pelletier J, Welsch DJ, Pelletier JP (2001). Metalloproteases and inhibitors in arthritic diseases. Best Pract Res Clin Rheumatol.

[B8] Murphy G, Knauper V, Atkinson S, Butler G, English W, Hutton M, Stracke J, Clark I (2002). Matrix metalloproteinases in arthritic disease. Arthritis Res.

[B9] Mengshol JA, Mix KS, Brinckerhoff CE (2002). Matrix metalloproteinases as therapeutic targets in arthritic diseases: bull's-eye or missing the mark?. Arthritis Rheum.

[B10] Parks WC, Wilson CL, Lopez-Boado YS (2004). Matrix metalloproteinases as modulators of inflammation and innate immunity. Nat Rev Immunol.

[B11] Rutter JL, Mitchell TI, Buttice G, Meyers J, Gusella JF, Ozelius LJ, Brinckerhoff CE (1998). A single nucleotide polymorphism in the matrix metalloproteinase-1 promoter creates an Ets binding site and augments transcription. Cancer Res.

[B12] Price SJ, Greaves DR, Watkins H (2001). Identification of novel, functional genetic variants in the human matrix metalloproteinase-2 gene: role of Sp1 in allele-specific transcriptional regulation. J Biol Chem.

[B13] Ye S, Eriksson P, Hamsten A, Kurkinen M, Humphries SE, Henney AM (1996). Progression of coronary atherosclerosis is associated with a common genetic variant of the human stromelysin-1 promoter which results in reduced gene expression. J Biol Chem.

[B14] Jormsjo S, Whatling C, Walter DH, Zeiher AM, Hamsten A, Eriksson P (2001). Allele-specific regulation of matrix metalloproteinase-7 promoter activity is associated with coronary artery luminal dimensions among hypercholesterolemic patients. Arterioscler Thromb Vasc Biol.

[B15] Zhang B, Ye S, Herrmann SM, Eriksson P, de Maat M, Evans A, Arveiler D, Luc G, Cambien F, Hamsten A (1999). Functional polymorphism in the regulatory region of gelatinase B gene in relation to severity of coronary atherosclerosis. Circulation.

[B16] Yoon S, Kuivaniemi H, Gatalica Z, Olson JM, Buttice G, Ye S, Norris BA, Malcom GT, Strong JP, Tromp G (2002). MMP13 promoter polymorphism is associated with atherosclerosis in the abdominal aorta of young black males. Matrix Biol.

[B17] Ladenvall P, Wall U, Jern S, Jern C (2000). Identification of eight novel single-nucleotide polymorphisms at human tissue-type plasminogen activator (t-PA) locus: association with vascular t-PA release in vivo. Thromb Haemost.

[B18] Dawson SJ, Wiman B, Hamsten A, Green F, Humphries S, Henney AM (1993). The two allele sequences of a common polymorphism in the promoter of the plasminogen activator inhibitor-1 (PAI-1) gene respond differently to interleukin-1 in HepG2 cells. J Biol Chem.

[B19] North BV, Curtis D, Sham PC (2005). Application of logistic regression to case-control association studies involving two causative loci. Hum Hered.

[B20] Erdfelder E, Faul F, Buchner A (1996). GPower: A general power analysis program. Behav Res Methods Instrum Comput.

[B21] Abecasis GR, Cookson WO (2000). GOLD – graphical overview of linkage disequilibrium. Bioinformatics.

[B22] Qin ZS, Niu T, Liu JS (2002). Partition-ligation-expectation-maximization algorithm for haplotype inference with single-nucleotide polymorphisms. Am J Hum Genet.

[B23] Sham PC, Curtis D (1995). Monte Carlo tests for associations between disease and alleles at highly polymorphic loci. Ann Hum Genet.

[B24] Ioannidis JP, Ntzani EE, Trikalinos TA, Contopoulos-Ioannidis DG (2001). Replication validity of genetic association studies. Nat Genet.

[B25] Busso N, Hamilton JA (2002). Extravascular coagulation and the plasminogen activator/plasmin system in rheumatoid arthritis. Arthritis Rheum.

[B26] Sanchez-Pernaute O, Largo R, Calvo E, Alvarez-Soria MA, Egido J, Herrero-Beaumont G (2003). A fibrin based model for rheumatoid synovitis. Ann Rheum Dis.

[B27] So AK, Varisco PA, Kemkes-Matthes B, Herkenne-Morard C, Chobaz-Peclat V, Gerster JC, Busso N (2003). Arthritis is linked to local and systemic activation of coagulation and fibrinolysis pathways. J Thromb Haemost.

[B28] Saxne T, Lecander I, Geborek P (1993). Plasminogen activators and plasminogen activator inhibitors in synovial fluid. Difference between inflammatory joint disorders and osteoarthritis. J Rheumatol.

[B29] Busso N, Peclat V, So A, Sappino AP (1997). Plasminogen activation in synovial tissues: differences between normal, osteoarthritis, and rheumatoid arthritis joints. Ann Rheum Dis.

[B30] Yang YH, Carmeliet P, Hamilton JA (2001). Tissue-type plasminogen activator deficiency exacerbates arthritis. J Immunol.

[B31] Cook AD, Braine EL, Campbell IK, Hamilton JA (2002). Differing roles for urokinase and tissue-type plasminogen activator in collagen-induced arthritis. Am J Pathol.

[B32] Wolf AT, Medcalf RL, Jern C (2005). The t-PA -7351C>T enhancer polymorphism decreases Sp1 and Sp3 protein binding affinity and transcriptional responsiveness to retinoic acid. Blood.

[B33] Ladenvall P, Johansson L, Jansson JH, Jern S, Nilsson TK, Tjarnlund A, Jern C, Boman K (2002). Tissue-type plasminogen activator -7,351C/T enhancer polymorphism is associated with a first myocardial infarction. Thromb Haemost.

[B34] Jannes J, Hamilton-Bruce MA, Pilotto L, Smith BJ, Mullighan CG, Bardy PG, Koblar SA (2004). Tissue plasminogen activator -7351C/T enhancer polymorphism is a risk factor for lacunar stroke. Stroke.

[B35] Gruber BL, Sorbi D, French DL, Marchese MJ, Nuovo GJ, Kew RR, Arbeit LA (1996). Markedly elevated serum MMP-9 (gelatinase B) levels in rheumatoid arthritis: a potentially useful laboratory marker. Clin Immunol Immunopathol.

[B36] Ahrens D, Koch AE, Pope RM, Stein-Picarella M, Niedbala MJ (1996). Expression of matrix metalloproteinase 9 (96-kd gelatinase B) in human rheumatoid arthritis. Arthritis Rheum.

[B37] Konttinen YT, Ainola M, Valleala H, Ma J, Ida H, Mandelin J, Kinne RW, Santavirta S, Sorsa T, Lopez-Otin C, Takagi M (1999). Analysis of 16 different matrix metalloproteinases (MMP-1 to MMP-20) in the synovial membrane: different profiles in trauma and rheumatoid arthritis. Ann Rheum Dis.

[B38] Schurigt U, Stopfel N, Huckel M, Pfirschke C, Wiederanders B, Brauer R (2005). Local expression of matrix metalloproteinases, cathepsins, and their inhibitors during the development of murine antigen-induced arthritis. Arthritis Res Ther.

[B39] Itoh T, Matsuda H, Tanioka M, Kuwabara K, Itohara S, Suzuki R (2002). The role of matrix metalloproteinase-2 and matrix metalloproteinase-9 in antibody-induced arthritis. J Immunol.

[B40] McQuibban GA, Gong JH, Tam EM, McCulloch CA, Clark-Lewis I, Overall CM (2000). Inflammation dampened by gelatinase A cleavage of monocyte chemoattractant protein-3. Science.

[B41] McQuibban GA, Butler GS, Gong JH, Bendall L, Power C, Clark-Lewis I, Overall CM (2001). Matrix metalloproteinase activity inactivates the CXC chemokine stromal cell-derived factor-1. J Biol Chem.

[B42] Price SJ, Greaves DR, Watkins H (2001). Identification of novel, functional genetic variants in the human matrix metalloproteinase-2 gene: role of Sp1 in allele-specific transcriptional regulation. J Biol Chem.

[B43] Yu C, Zhou Y, Miao X, Xiong P, Tan W, Lin D (2004). Functional haplotypes in the promoter of matrix metalloproteinase-2 predict risk of the occurrence and metastasis of esophageal cancer. Cancer Res.

[B44] Yu C, Pan K, Xing D, Liang G, Tan W, Zhang L, Lin D (2002). Correlation between a single nucleotide polymorphism in the matrix metalloproteinase-2 promoter and risk of lung cancer. Cancer Res.

[B45] Miao X, Yu C, Tan W, Xiong P, Liang G, Lu W, Lin D (2003). A functional polymorphism in the matrix metalloproteinase-2 gene promoter (-1306C/T) is associated with risk of development but not metastasis of gastric cardia adenocarcinoma. Cancer Res.

[B46] Zhou Y, Yu C, Miao X, Tan W, Liang G, Xiong P, Sun T, Lin D (2004). Substantial reduction in risk of breast cancer associated with genetic polymorphisms in the promoters of the matrix metalloproteinase-2 and tissue inhibitor of metalloproteinase-2 genes. Carcinogenesis.

[B47] Lin SC, Lo SS, Liu CJ, Chung MY, Huang JW, Chang KW (2004). Functional genotype in matrix metalloproteinases-2 promoter is a risk factor for oral carcinogenesis. J Oral Pathol Med.

[B48] Xu E, Lai M, Lv B, Xing X, Huang Q, Xia X (2004). A single nucleotide polymorphism in the matrix metalloproteinase-2 promoter is associated with colorectal cancer. Biochem Biophys Res Commun.

[B49] Holla LI, Fassmann A, Vasku A, Goldbergova M, Beranek M, Znojil V, Vanek J, Vacha J (2005). Genetic variations in the human gelatinase A (matrix metalloproteinase-2) promoter are not associated with susceptibility to, and severity of, chronic periodontitis. J Periodontol.

[B50] Mercado FB, Marshall RI, Bartold PM (2003). Inter-relationships between rheumatoid arthritis and periodontal disease. A review. J Clin Periodontol.

